# Roles of the Polymerase-Associated Protein Genes in Newcastle Disease Virus Virulence

**DOI:** 10.3389/fmicb.2017.00161

**Published:** 2017-02-06

**Authors:** Xiao-hui Yu, Jin-long Cheng, Jia Xue, Ji-hui Jin, Yang Song, Jing Zhao, Guo-zhong Zhang

**Affiliations:** Key Laboratory of Animal Epidemiology and Zoonosis, Ministry of Agriculture, College of Veterinary Medicine, China Agricultural UniversityBeijing, China

**Keywords:** genotype VII, Newcastle disease virus, virulence, growth kinetics, replication, reverse genetics

## Abstract

The virulence of Newcastle disease virus varies greatly and is determined by multiple genetic factors. In this study, we systematically evaluated the roles of the polymerase-associated (NP, P and L) protein genes in genotype VII NDV virulence after confirming the envelope-associated (F and HN) proteins contributed greatly to NDV virulence. The results revealed that the polymerase-associated protein genes individually had certain effect on virulence, while transfer of these three genes in combination significantly affected the chimeric virus virulence, especially when the L gene was involved. These results indicated that the L protein was a major contributor to NDV virulence when combined with the homologous NP and P proteins. We also investigated viral RNA synthesis using NDV minigenome systems to assess the interaction between the NP, P, and L proteins, which showed that the activity of the polymerase-associated proteins were directly related to viral RNA transcription and replication.

## Introduction

Newcastle disease (ND) is a severe infectious disease of poultry caused by the Newcastle disease virus (NDV) that affects more than 250 species of birds. NDV belongs to the genus *Avulavirus* within the family *Paramyxoviridae* and its genome is a non-segmented single-stranded RNA molecule of 15,186, 15,192, or 15,198 nucleotides (nt) in length (Mayo, 2002; Paldurai et al., 2014). The genome consisting of six genes in the order 3′-NP-P-M-F-HN-L-5′ encodes six structural proteins: nucleoprotein (NP), phosphoprotein (P), matrix protein (M), fusion protein (F), hemagglutinin-neuraminidase (HN), and large polymerase protein (L). Two additional non-structural proteins, V and W, are encoded by means of RNA editing during P gene transcription (Steward et al., 1993).

Based on their pathogenicity in chickens, NDV strains can be categorized into three different pathotypes: lentogenic (low virulence), mesogenic (moderate virulence) and velogenic (high virulence) (Alexander, 2003). The virulence of NDV is a complex trait that is determined by multiple factors, the cleavage site of the F protein being the major virulence determinant with other factors being critically involved (Alexander, 2003; Estevez et al., 2007; Rout and Samal, 2008; Dortmans et al., 2010; Kim et al., 2011; Paldurai et al., 2014). However, many studies have indicated that the contribution of these factors to NDV virulence may be associated with the particular virus strain used and the findings from these reports have not always been consistent or conclusive (Estevez et al., 2007; Rout and Samal, 2008; Dortmans et al., 2009, 2010; Wakamatsu et al., 2009; Paldurai et al., 2014).

Newcastle disease viruses can be classified into different genotypes based on the sequence and phylogenetic analysis of the F protein gene. Since the 1990s, genotype VII NDV strains have frequently been reported in many regions including Asia, Europe, the Middle East and South Africa (Herczeg et al., 1999; Bogoyavlenskiy et al., 2009; Berhanu et al., 2010; Rui et al., 2010), indicating that genotype VII NDV is the predominant virus currently circulating in the world and constituting the fourth panzootic strain of NDV. Previous studies have revealed that genotype VII NDVs have some new features and a higher pathogenicity in chickens compared with other virulent strains, although they have the same F protein cleavage site (Hu et al., 2012, 2015; Zhang et al., 2014; Kai et al., 2015). However, whether the contribution of the viral proteins of genotype VII strains to the virulence and pathogenicity of NDV is the same as in other strains remains unclear.

In this study, we evaluated the contributions to genotype VII NDV virulence of the envelope-associated protein genes (F and HN) in combination, as well as the polymerase-associated protein genes (NP, P and L) individually or in combination. In addition to the two parent strains (a lentogenic genotype II strain, LaSota, and a velogenic genotype VII strain, SG10), we recovered 16 chimeric viruses using reverse genetics. The pathogenicity of the chimeric viruses in 1-day-old chicks as well as in 4-week-old chickens, the replication kinetics in DF-1 cells and chicken embryos, and the RNA synthesis in NDV minigenome systems were analyzed. Our results reveal that both the envelope-associated and polymerase-associated proteins contribute to the virulence of genotype VII NDV, and the activity of the polymerase-associated proteins is directly related to viral RNA synthesis.

## Materials and Methods

### Animal and Ethics Statement

Specific-pathogen-free (SPF) chicken eggs and chickens were purchased from Merial Vital Laboratory Animal Technology Co., Ltd (Beijing, China). All birds were kept in isolators at the China Agricultural University throughout the experiments and the animal rearing facilities were approved by the Administration Committee of Laboratory Animals under the auspices of the Beijing Association for Science and Technology (approval ID SYXK [Jing] 2013-0013).

The study was carried out in strict accordance with the Guidance for the Care and Use of Laboratory Animals formulated by the Ministry of Science and Technology of the People’s Republic of China. The experimental protocol, including the possibility of animal death without euthanasia, was specifically considered and approved by the Animal Welfare and Ethical Censor Committee at China Agricultural University (CAU approval number 1603–05). All surgery was performed under sodium pentobarbital anesthesia, and all efforts were made to minimize suffering.

### Cells and Viruses

A chicken embryo fibroblast cell line (DF-1) was grown in Dulbecco’s modified Eagle’s medium (DMEM) (Gibco, Grand Island, NY) containing 10% (v/v) fetal bovine serum (FBS) (Gibco) and maintained in DMEM containing 5% FBS. Baby hamster kidney (BHK-21) cells stably expressing T7 RNA polymerase (BSR T7 cells) were grown in DMEM containing 10% FBS and 1 mg/ml G418 (Invitrogen, Carlsbad, CA, USA). Both cell lines were grown at 37°C in an incubator (Thermo Forma, Marietta, OH, USA) under 5% CO_2_. NDV strain SG10 was isolated from an outbreak of ND in chickens and identified as velogenic genotype VII virus with the intracerebral pathogenicity index (ICPI) of 1.79 and the mean death time (MDT) of 45 h (h) (Liu et al., 2015). NDV strain SG10 and the lentogenic genotype II strain LaSota (ICPI = 0, MDT > 120 h) were purified using the endpoint dilution method and propagated in 9-day-old SPF embryonated chicken eggs via allantoic cavity inoculation.

### Construction of Full-Length Chimeric SG10/LaSota Antigenomic cDNAs

**FIGURE 1:**
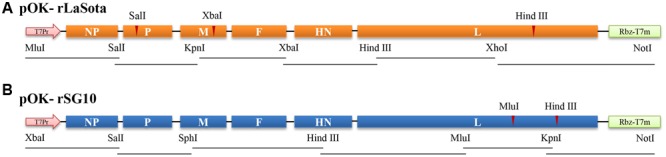
**Construction of full-length antigenomic cDNAs of rLaSota (A)** and rSG10 **(B)**. The seven unique restriction sites used in constructing the antigenomic cDNAs and for exchanging genes are shown for lentogenic genotype II strain rLaSota **(A)** and velogenic genotype VII NDV strain rSG10 **(B)**.

The construction of full-length antigenomic cDNA of NDV strain SG10 (rSG10) has been described previously (**Figure 1**) (Liu et al., 2015). To construct the recombinant LaSota cDNA clone, six fragments spanning the full-length LaSota cDNA were generated by reverse transcription-polymerase chain reaction (RT-PCR) of RNA that had been purified from the allantoic fluid of infected eggs. The six fragments were sequentially cloned into the pOK12 vector through seven restriction sites (*Mlu*I, *Sal*I, *Kpn*I, *Xba*I, *Hind*III, *Xho*I, and *Not*I). The leader region was preceded by a T7 RNA polymerase promoter that initiated the encoded antigenomic RNA with three G residues at its 5′ end. The trailer region was flanked by a hepatitis delta virus ribozyme sequence that cleaved after the last NDV-specific residue (**Figure 1**). Of the seven restriction sites in the NDV sequence, the *Mlu*I and the *Not*I restriction sites were introduced during RT-PCR and the others occurred naturally on the genome. In addition, in order for the restriction sites to be unique, the naturally occurring *Sal*I, *Xba*I and *Hind*III sites in the P, M and L open reading frames (ORFs) were eliminated without changing the amino acid coding to act as genetic markers. The constructed plasmid was designated pOK-rLaSota and was confirmed to be identical to the biological virus by complete nucleotide sequencing.

The F and HN ORFs of NDV strain rSG10 were inserted in combination into a full-length antigenomic cDNA of strain rLaSota in place of the original NDV F and HN ORFs using the presence of unique restriction enzyme sites and the Seamless Assembly Cloning Kit (Invitrogen). First, the full-length antigenome of rLaSota was digested with *Kpn*I and *Hind*III to generate a single DNA fragment without the F and HN genes. Second, the F and HN ORFs of rSG10 were engineered by PCR amplification as two DNA fragments each flanked by 5′ and 3′ UTRs of the respective rLaSota F or HN genes. To maintain the genome length as a multiple of six nucleotides, three extra nucleotides were introduced at the 3′UTR of the HN gene as necessary. The other three DNA fragments with homologous ends, one extending from the *Kpn*I site to the F initiation codon, one from the F termination codon to the HN initiation codon and the other from the HN termination codon to the *Hind*III site, were generated by PCR amplification with compatible primers. Finally, these six DNA fragments with overlapping homologous ends of various lengths were effectively joined together using the Cloning Kit. The complete F and HN ORFs of NDV strain LaSota were inserted in combination into the SG10 backbone in place of the corresponding SG10 sequence using the same strategy. The full-length rLaSota cDNA plasmid containing the rSG10 F and HN genes was designated rLaSGFHN, whereas the full-length cDNA plasmid of rSG10 containing the rLaSota F and HN genes was designated rSGLaFHN.

The NP, P, and L ORFs were reciprocally exchanged between the LaSota and SG10 antigenome cDNAs using the relevant restriction enzyme sites noted above and the Seamless Assembly Cloning Kit (Invitrogen). Seven chimeras based on a full-length antigenomic cDNA of rLaSota was constructed in the same strategy: rLaSGNP, rLaSGP, rLaSGL, rLaSGNPP, rLaSGNPL, rLaSGPL, and rLaSGNPPL, as well as a parallel set of seven constructs based on a full-length antigenomic cDNA of rSG10: rSGLaNP, rSGLaP, rSGLaL, rSGLaNPP, rSGLaNPL, rSGLaPL, and rSGLaNPPL. All the exchanged regions in the full-length cDNAs were sequenced by dideoxynucleotide sequencing to confirm the presence of the desired genes.

### Recovery of Virus from cDNA

The recombinant parental and chimeric viruses were recovered by co-transfection of each NDV chimeric full-length cDNA plasmid, along with helper plasmids expressing the NP, P, and L proteins, into BSR T7/5 cells, as previously described Liu et al. (2015). The respective helper plasmids of either LaSota or SG10 were used along with their respective full-length cDNAs to prevent potential heterologous recombination in the transfected cells. Four days after transfection, the cell culture supernatants and cell monolayers were harvested and used to recover the chimeric NDVs by injection into the allantoic cavities of 9-day-old SPF embryonated chicken eggs. Recovery of the virus was confirmed by hemagglutination (HA) assay using 1% chicken red blood cells (RBCs). Total RNA was extracted from NDV-positive allantoic fluid with TRIzol reagent (Invitrogen) according to the manufacturer’s instructions. The rLaSota parents were completely sequenced, while only the exchanged genes and flanking sequences of the chimeric derivatives were sequenced. No adventitious mutations were detected. Cells transfected with rLaSota chimeric cDNAs were maintained in medium supplemented with 5 μg/ml *N*-tosylphenylalanine chloromethyl ketone (TPCK)-treated trypsin (Sigma-Aldrich, St. Louis, MO, USA) as a source of exogenous protease (Madansky and Bratt, 1981).

### MDT and ICPI of the Recombinant Viruses

The pathogenicity of the chimeric viruses was determined by the MDT test in 9-day-old SPF embryonated chicken eggs and by the ICPI test in 1-day-old SPF chicks as described elsewhere (Cevik et al., 2004). Briefly, for the MDT test, a series of 10-fold dilutions of infected allantoic fluid was made in sterile phosphate buffered saline (PBS), and each dilution (0.1 ml) was inoculated into five 9-day-old eggs via the allantoic cavities route and incubated at 37°C. The eggs were examined once every 8 h for 7 days, and the time at which mortality of each embryo was first observed was recorded. The highest dilution yielding 100% mortality was considered to be the minimum lethal dose. The MDT was determined as the mean time (in hours) for the minimum lethal dose of virus to kill all the inoculated embryos. The MDT has been used to classify NDV strains as velogenic (taking less than 60 h to kill), mesogenic (taking 60–90 h to kill) or lentogenic (taking more than 90 h to kill). For the ICPI tests, for each virus 0.05 ml of a 1:10 dilution of fresh infective allantoic fluid was inoculated into 10 1-day-old SPF chicks via the intracerebral route. The birds were monitored for clinical symptoms and mortality once every 24 h for 8 days. At each observation, the birds were scored as follows: 0 if normal, 1 if sick and 2 if deceased. The ICPI is the mean of the score per bird per observation over the 8-day period. The pathotype definitions by the ICPI are as follows: virulent strains, 1.50–2.00; moderately virulent strains, 0.70–1.50; avirulent strains, 0.00–0.70.

### Virus Growth Kinetics

The growth kinetics of rLaSota and rSG10 and their chimeric viruses were determined under multiple-cycle growth conditions in DF-1 cells and in SPF chicken embryos. Duplicate wells of six-well plates containing DF-1 cells were infected with virus at a multiplicity of infection (MOI) of 0.01. After 1 h of adsorption, the cells were washed with DMEM and then covered with DMEM containing 5% FBS and placed at 37°C under 5% CO_2_ in an incubator. The medium of the cells infected with rLaSota or its chimeric viruses was supplemented with 5 μg/ml TPCK-treated trypsin. Supernatant samples were collected and replaced with an equal volume of fresh medium at 12 h intervals until 72 h post-infection (hpi). Virus titers in the collected supernatants were quantified via limiting dilution in DF-1 cells using the endpoint method of Reed and Muench (1938) and expressed as the 50% tissue culture infective dose (TCID_50_).

To analyze the growth kinetics of chimeric viruses in chicken embryos, the allantoic cavities of 9-day-old SPF chicken embryos were inoculated with 10^6^ TCID_50_ of each virus per embryo. Three embryos were chilled at regular 12 h intervals until 72 hpi, allantoic fluid samples were harvested, and the titers (TCID_50_) of virus in the collected samples were determined by the method described above.

### Pathogenicity Assessment in Chickens

Two viruses, rSGLaNPPL and rLaSGNPPL, and their parent strains (rSG10 and rLaSota) were used to determine the pathogenicity in chickens. Four-week-old SPF white Leghorn chickens were assigned randomly to five groups of 20 birds each (10 for sampling and 10 for clinical observation) and each group was inoculated by the oculonasal route with either one of the four viruses or with 0.9% NaCl as the negative control. The target dose of the inoculum was 10^6^ 50% egg infectious dose (EID_50_) of virus per bird. The birds were monitored daily for clinical signs for 14 dpi. Two birds were euthanized daily from days 1–5 pi for detection of gross lesions, and samples were collected into two parts. One part (trachea, lung, brain, proventriculus, cecal tonsil, liver, spleen, and duodenum) was used for virus titration and the other part (brain, spleen, lung, proventriculus, trachea and cecal tonsil) was fixed in 10% buffered formalin for histopathology. Necropsies were carried out, and external and internal abnormalities were recorded.

For virus titration, the tissue samples were homogenized in PBS containing antibiotics and virus titers were measured in DF-1 cells. The cleared tissue homogenates were serially diluted 10-fold and inoculated in DF-1 cells. At 72 hpi, cells were fixed with cold methanol and incubated with hyper-immune anti-NDV serum at 37°C for 1 h. Then the cells were washed three times with PBS and incubated with the secondary antibody, goat anti-chicken IgG-FITC (Sigma–Aldrich), at 37°C for 1 h. The virus titers were determined as TCID_50_ per gram using the Reed and Muench method (Reed and Muench, 1938).

For histopathology, tissue samples were fixed in 10% neutral buffered formalin for 48 h. All sampled tissues were routinely embedded in paraffin and 5-μm sections were cut for hematoxylin and eosin staining and examined for lesions using light microscopy. The tissue sections were scored according to the severity of the observed lesions. The absence of injury was classified as -, while mild, moderate and severe injuries were classified as +, ++, and +++, respectively.

### NDV Minigenome System and Dual Luciferase Assay

To detect whether viral RNA synthesis correlated with the intrinsic activity of the polymerase-associated proteins, two synthetic DNAs containing the T7 RNA polymerase promoter and the firefly luciferase (FLuc) gene (in an antisense orientation) flanked at the 5′ side by the trailer region and at the 3′ side by the leader region (LaSota and SG10), followed by the hepatitis delta virus ribozyme and the transcription termination signal, were synthesized and cloned into plasmid pGL3-Basic with *Kpn*I and *Xba*I (Nishio et al., 2001). The constructed minigenome plasmids in which the number of nucleotides from the start of the trailer sequence to the end of the leader sequence was a multiple of six, were designated pLaSota-FLuc and pSG10-FLuc.

The BSR T7 cells were seeded in 24-well culture plates. The cells were cotransfected with either pLaSota-FLuc or pSG10-FLuc (340 ng) and helper plasmids expressing NP (340 ng), P (170 ng), and L (170 ng) proteins originating either from the LaSota strain or from the SG10 strain as well as a Renilla luciferase reporter gene (RLuc, 10 ng). At 20–22 hpi, the cells were washed in PBS once, and 100 μL passive lysis buffer (Promega, Madison, WI, USA) was added. Cells were vigorously mixed for 15 min, and 20 μL of lysate from each well were used for dual luciferase assay by dual luciferase assay kit (Promega). The relative luciferase activity unit was defined as the ratio of FLuc to RLuc activities (Dortmans et al., 2010). Six separate experiments were performed, with luciferase expression being measured in triplicate in each experiment.

### Statistical Analyses

All statistical analyses were performed using the unpaired *t-*test in GraphPad Prism Software Version 6.0 (GraphPad Software Inc., San Diego, CA, USA). Statistically significant differences between experimental groups were determined using the analysis of variance (ANOVA) method. The significance was considered as follows: significant, *p* = 0.01–0.05 (^∗^); very significant, *p* = 0.001–0.01 (^∗∗^); extremely significant, *p* = 0.0001–0.001 (^∗∗∗^).

## Results

### Recovery of Recombinant Chimeric Viruses

A cDNA clone encoding the antigenome of strain LaSota (rLaSota) was constructed from six cDNA segments that were synthesized by RT-PCR from virion-derived genomic RNA (**Figure 1**). The virulence of rLaSota was evaluated by the ICPI test in 1-day-old SPF chicks. The ICPI value of rLaSota was 0, which was the same as its biological parent LaSota (date not shown). These results showed that the cDNA-derived strain rLaSota and its parent had the same biological characteristics.

To determine the role of the viral surface glycoproteins in NDV virulence, the complete ORFs of the F and HN genes in combination were reciprocally exchanged between full-length antigenomic cDNAs of the velogenic genotype VII NDV strain rSG10 and the low-virulence genotype II strain rLaSota. Sequence analysis of the chimeric cDNAs confirmed the intended gene exchanges and the absence of any undesired mutations. The two recovered recombinant viruses were named rSGLaFHN and rLaSGFHN.

To evaluate the contributions of the polymerase-associated proteins (NP, P, and L) to the virulence of genotype VII NDV, the genes encoding these proteins were exchanged between strains rLaSota and rSG10 using reverse genetics. Fourteen chimeric viruses were recovered. Seven chimeric viruses were derivatives of strain rLaSota in which the NP, P and L genes were replaced individually or in combination with those of rSG10. Seven chimeric viruses were derivatives of rSG10, in which the NP, P and L ORFs were replaced individually or in combination with those of rLaSota. All chimeric viruses were amplified by RT-PCR and sequenced, which confirmed the correct structure of each exchange and a lack of adventitious mutations.

### Pathogenicity of Chimeric Viruses in Chicken Embryos and Day-Old Chickens

To determine the contributions to genotype VII NDV virulence of the F and HN protein genes in combination, the virulence of the two chimeric viruses along with their respective parental viruses was evaluated by determining the ICPI and MDT tests. The respective MDT values of parental rSG10 and rLaSota were 57 h and >120 h in embryonated eggs, and they had ICPI values of 1.86 and 0.00, respectively (**Table 1**). These values were consistent with the designations of rSG10 as velogenic and rLaSota as avirulent. Replacement of the envelope genes (F and HN) of the rSG10 strain in combination with those of the rLaSota strain (rSGLaFHN) resulted in a significant decrease in virulence, the MDT and ICPI values being >120 h and 0.86, respectively. Derivatives of rLaSota bearing the envelope protein genes (F and HN) of rSG10 (rLaSGFHN) in combination showed an obvious increased virulence with MDT and ICPI values of 79 h and 1.49 respectively (**Table 1**). The results indicated that exchange of these envelope-associated protein genes in combination between the low-virulence strain LaSota and the highly virulent genotype VII strain SG10 had a marked effect on the viral virulence.

**Table 1:** Intracerebral pathogenicity index (ICPI) scores and MDT values for parental rLaSota and rSG10 and their envelope-associated protein gene chimeras.

Virus	ICPI score^a^	MDT (h)^b^
rLaSota	0.00	>120
rLaSGFHN	1.49ˆ*	79ˆ*
		
rSG10	1.86	57
rSGLaFHN	0.86ˆ*	>120ˆ*

*^a^Pathotype definitions by ICPI: virulent strains, 1.50–2.00; moderately virulent strains, 0.70–1.50; avirulent strains, 0.00–0.70. ^b^Pathotype definitions by MDT: virulent strains, <60 h; moderately virulent strains, 60–90 h; avirulent strains, >90 h. Asterisks indicate the statistical significance of the ICPI score and MDT of a chimeric virus compared to the parental virus of that group. *P*-values were calculated based on a two-tailed, unpaired *t*-test (95% confidence levels). ^∗^*p* = 0.01–0.05, significant.*

We further examined the roles of the polymerase-associated proteins genes (NP, P and L) in NDV virulence. Derivatives of rSG10 bearing the polymerase-associated protein genes of rLaSota had respective MDT/ICPI values as follows: rSGLaNP, 72 h/1.78; rSGLaP, 52 h/1.89; rSGLaL, 83 h/1.68; rSGLaNPP, 58 h/1.90; rSGLaNPL, 86 h/1.48; rSGLaPL, 84 h/1.40; and rSGLaNPPL, 89 h/1.32 (**Table 2**). Derivatives of rLaSota bearing the polymerase-associated protein genes of rSG10 had respective MDT/ICPI values as follows: rLaSGNP, >120 h/0.00; rLaSGP, >120 h/0.00; rLaSGL, >120 h/0.00; rLaSGNPP, >120 h/0.34; rLaSGNPL, >120 h/0.14; rLaSGPL, >120 h/0.12; and rLaSGNPPL, >120 h/0.63 (**Table 2**). The results indicated that the individual replacement of the polymerase-associated genes between the SG10 strain and LaSota strain did not affect the virulence of NDV except for the rSGLaL. The simultaneous replacement of the three polymerase-associated genes (NP + P + L) had an evident effect on viral virulence, as shown by a decrease in the ICPI value from 1.86 for rSG10 to 1.32 for rSGLaNPPL and an increase in the ICPI value from 0.00 for rLaSota to 0.63 for rLaSGNPPL (**Table 2**).

**Table 2:** Intracerebral pathogenicity index scores and MDT values for parental rLaSota and rSG10 and their polymerase-associated protein gene chimeras.

Virus	ICPI score^a^	MDT (h)^b^
rLaSota	0.00	>120
rLaSGNP	0.00	>120
rLaSGP	0.00	>120
rLaSGL	0.00	>120
rLaSGNPP	0.34ˆ**	>120
rLaSGNPL	0.14ˆ*	>120
rLaSGPL	0.12ˆ*	>120
rLaSGNPPL	0.63ˆ***	>120
rSG10	1.86	57
rSGLaNP	1.78	72^∗∗^
rSGLaP	1.89	52
rSGLaL	1.68ˆ**	83^∗∗∗^
rSGLaNPP	1.90	58
rSGLaNPL	1.48ˆ***	86^∗∗∗^
rSGLaPL	1.40ˆ***	84^∗∗∗^
rSGLaNPPL	1.32ˆ***	89^∗∗∗^

*^a^Pathotype definitions by ICPI: virulent strains, 1.50–2.00; moderately virulent strains, 0.70–1.50; avirulent strains, 0.00–0.70. ^b^Pathotype definitions by MDT: virulent strains, <60 h; moderately virulent strains, 60–90 h; avirulent strains, >90 h. Asterisks indicate the statistical significance of the ICPI score and MDT of a chimeric virus compared to the parental virus of that group. *P*-values were calculated based on a two-tailed, unpaired t test (95% confidence levels). ^∗^*p* = 0.01–0.05, significant; ^∗∗^*p* = 0.001–0.01, very significant; ^∗∗∗^*p* = 0.0001–0.001, extremely significant.*

### Growth Characteristics of Chimeric Viruses in DF-1 Cells

**FIGURE 2:**
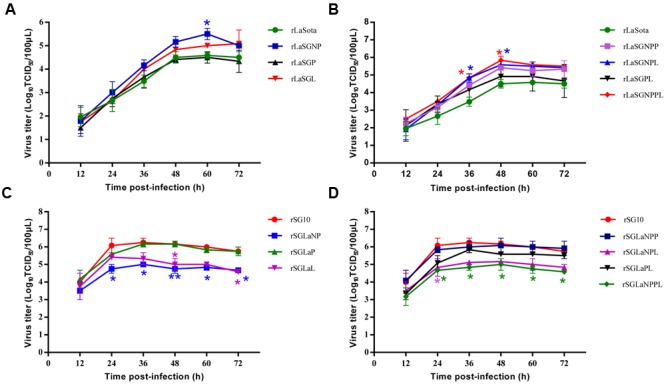
***In vitro* growth characterization of parental and chimeric viruses in DF-1 cells. (A,B)** rLaSota-based chimeras in which the polymerase-associated protein genes NP, P, and L were replaced by their counterparts from rSG10 individually **(A)** or in combination **(B)**. **(C,D)** rSG10-based chimeras in which the polymerase-associated protein genes NP, P, and L were replaced by their counterparts from rLaSota individually **(C)** or in combination **(D)**. Asterisks indicate the test of significance of the virus titer of a chimeric virus compared to the parental virus; *p-*values were calculated based on a two-tailed, unpaired *t-*test (95% confidence levels). ^∗^*p* = 0.01–0.05, significant; ^∗∗^*p* = 0.001–0.01, very significant.

The growth kinetics of 14 chimeric viruses in which the NP, P, and L genes were replaced individually or in combination between the low-virulence strain LaSota and the highly virulent genotype VII NDV strain SG10, along with their respective parental viruses, were compared using multicycle growth curves in DF-1 cells. When the growth of strains rLaSGNP, rLaSGP and rLaSGL was compared with that of the parental rLaSota, no significant growth differences were observed (*p* > 0.05) except for rLaSGNP which showed an obvious increase at 60 hpi (*p* < 0.05) (**Figure 2A**). However, the chimeric viruses rLaSGNPL and rLaSGNPPL, in which the NP + L or NP + P + L genes of the rLaSota virus in combination were replaced with those of the rSG10 virus, showed much higher virus titre than the parental rLaSota virus at 36 h and 48 hpi (*p* < 0.05) (**Figure 2B**). These results indicated that, among the polymerase-associated protein, the NP and L proteins had an obvious influence on the level of virus replication and this influence was greatest when induced by the interaction of homologous NP, P, and L proteins.

Some constructs of rSG10 bearing rLaSota-derived NP, P, and L genes, alone or in combination, showed a decreased replication compared with parental rSG10. The chimera rSGLaNP showed the lowest replication from 24 hpi to the end of the observation period (*p* < 0.05 or 0.01), while the chimera rSGLaL showed the lowest replication at 48 hpi (*p* < 0.05) (**Figure 2C**). The chimeric virus rSGLaNPL, in which the SG10 NP and L genes in combination were replaced with those of the LaSota virus, showed decreased replication at 24 hpi (*p* < 0.05), while another chimeric virus rSGLaNPPL also showed significantly decreased replication from 24 hpi to the end of the observation period (*p* < 0.05) (**Figure 2D**).

### Growth Characteristics of Chimeric Viruses in Chicken Embryos

**FIGURE 3:**
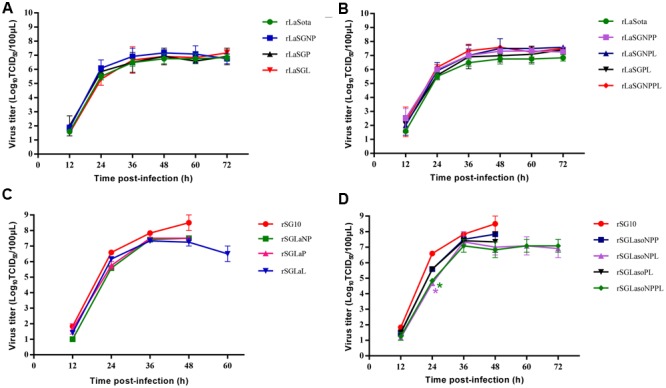
***In vivo* growth characterization of parental and chimeric viruses in 9-day-old chicken embryos. (A,B)** rLaSota-based chimeras in which the polymerase-associated protein genes NP, P, and L were replaced by their counterparts from rSG10 individually **(A)** or in combination **(B)**. **(C,D)** rSG10-based chimeras in which the polymerase-associated protein genes NP, P, and L were replaced by their counterparts from rLaSota individually **(C)** or in combination **(D)**. Asterisks indicate the test of significance of the virus titer of a chimeric virus compared to the parental virus; *p* values were calculated based on a two-tailed, unpaired *t-*test (95% confidence levels). ^∗^*p* = 0.01–0.05, significant.

The *in vivo* growth characteristics of the 14 chimeric viruses and their parental viruses were further evaluated in 9-day-old SPF chicken embryos (**Figure 3**). For chimeric viruses with LaSota backbone, rLaSGNP, rLaSGP and rLaSGL grew at the same rate as their parental rLaSota virus. The other chimeric viruses, of which the rSG10 virus-derived NP + L, NP + P, or NP + P + L genes in combination were replaced with those of the rLaSota virus, showed a slightly increased growth compared to the parental rLaSota (**Figures 3A,B**). This indicated that the presence of the polymerase-associated genes (NP, P, and L) of rSG10 virus individually in the rLaSota backbone did not alter the replication of chimeric viruses, while the combinations had a slight effect in 9-day-old chicken embryos. For the chimeric viruses with SG10 backbone, rSGLaL showed decreased replication compared to the parental rSG10 virus at 48 hpi, while the embryos infected with rSG10, rLaSGNP and rLaSGP had all died at this time. The chimeric viruses rSGLaNPL and rSGLaNPPL showed a significantly decreased replication at 24 hpi (*p* < 0.05) (**Figures 3C,D**). These results indicated that changing the NP or P gene did not alter the replication of the chimeric viruses compared with that of their parental viruses, except that rSGLaL, rSGLaNPL and rSGLaNPPL showed decreased replication at an early or later stage of infection.

### Replication and Pathogenicity of Chimeric Viruses in 4-week-old Chickens

Based on previous results, the pathogenicity of two chimeric viruses (rSGLaNPPL and rLaSGNPPL) and their parental viruses (rSG10 and rLaSota) were evaluated further in 4-week-old SPF chickens by inoculating each bird with 10^6^ EID_50_ of virus through the natural route of infection. No obvious clinical signs were observed in any of the birds inoculated with rLaSota, and slight punctate hemorrhages and catarrhal exudates were observed in the throat and trachea at days 4–5 pi (**Table 3**). Birds inoculated with rLaSGNPPL exhibited slight depression, punctate hemorrhages and catarrhal exudates in the throat and trachea, and hemorrhages and enlargement of the thymus at 4–5 dpi. The pathogenicity of the rSGLaNPPL and its parental virus differed greatly, the birds infected with rSGLaNPPL developing mild illness with moderate lesions and no mortalities, whereas the birds infected with rSG10 developed severe illness with severe lesions by days 3–5 pi and a 100% mortality by days 5.

**Table 3:** Summary of clinical signs and lesions in 4-week-old chickens infected with 0.9%NaCl, rLaSota, rLaSGNPPL, rSGLaNPPL, and rSG10.

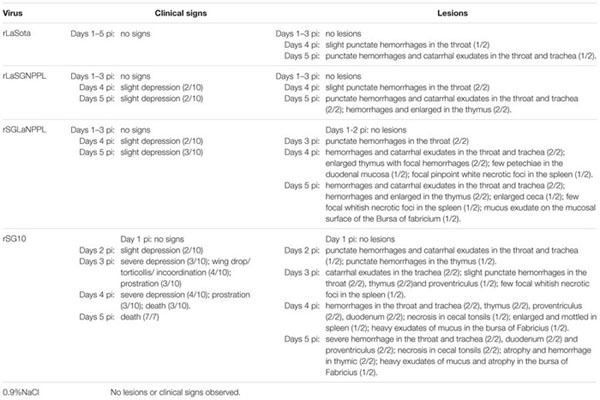

**FIGURE 4:**
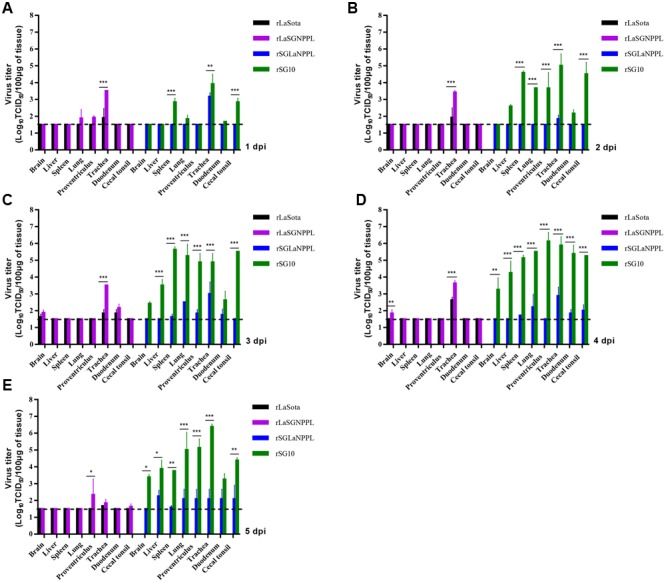
**Replication of parental and chimeric viruses in 4-week-old chickens.** Birds were inoculated with 10^6^ EID_50_ of rSG10, rLaSota, rLaSGNPPL or rSGLaNPPL through the natural route of infection, sacrificed at 1–5 dpi, and the indicated tissues collected and virus titers determined by indirect immunofluorescence in DF-1 cells. Asterisks indicate the test of significance of the virus titer of a chimeric virus compared with the parental virus; *p-*values were calculated based on a two-tailed, unpaired *t-*test (95% confidence levels). ^∗^*p* = 0.01–0.05, significant; ^∗∗^*p* = 0.001–0.01, very significant; ^∗∗∗^*p* = 0.0001–0.001, extremely significant.

Two chickens from each group were sacrificed daily on days 1–5 pi for virus titration by indirect immunofluorescence in DF-1 cells (**Figure 4**). The results showed that rLaSota could replicate in certain organs, such as trachea and occasionally in brain or duodenum. While rLaSGNPPL showed increased replication ability, and the virus replicated to moderate or high titers in many of the sampled tissues, such as proventriculus, brain, lung, duodenum and cecal tonsil and especially in trachea, the replication ability showed significant differences (*p* < 0.001) compared to rLaSota. For rSG10 and rSGLaNPPL, all sampled tissues in the rSG10-inoculated birds showed high viral titers from 3 dpi. The virus titers in most of the sampled tissues were significantly lower in rSGLaNPPL-inoculated birds than in birds inoculated with the parental rSG10 (*p* < 0.001), indicating a decreased replication ability of rSGLaNPPL compared with the parental virus.

**FIGURE 5:**
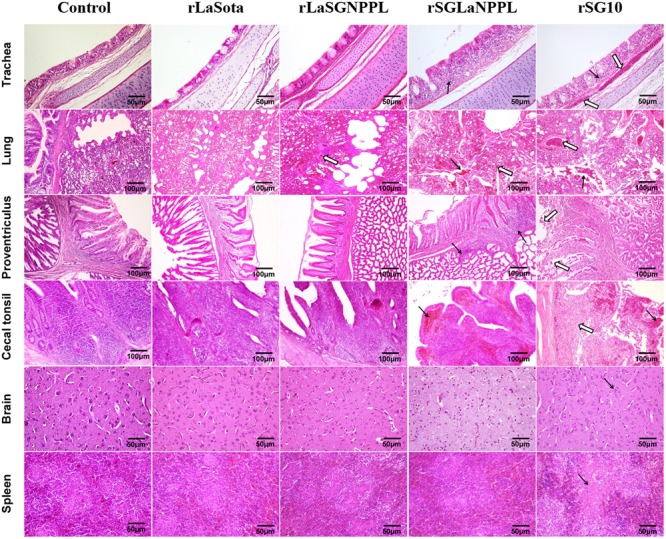
**Tissue histopathology of inoculated 4-week-old chickens.** Chickens were infected oculonasally with parental rSG10, parental rLaSota or their chimeric viruses (rLaSGNPPL and rSGLaNPPL). Birds were sacrificed at 4 dpi and tissue was fixed with formalin, sectioned and stained with hematoxylin and eosin. The trachea had lymphocytic infiltration of the lamina propria (black arrows) and submucosal congestion (empty arrows) of the tracheal mucosa. The lung had hemorrhage, RBC infiltration in both the lung and bronchi (black arrows) and congestion (empty arrows). The proventriculus had infiltration of lymphocytes in the mucous membranes of the nipple and the glands (black arrows) and severe mucosal epithelial shedding (empty arrows). The cecal tonsils indicated congestion of the lamina propria (black arrows) and necrosis and shedding of lymphocytic cells of the mucosa (empty arrows). The brain indicated increased and gathered microglial cells (black arrows). The spleen had necrosis and focal cellular necrosis formation (black arrows).

Two chickens from each group were sacrificed at days 4 pi for histopathological analysis (**Figure 5**; **Table 4**). For the chimera rLaSGNPPL and its parental virus rLaSota, no obvious histological changes were observed in sampled tissues except for focal congestion in the lungs of the group inoculated with rLaSGNPPL (empty arrows) (**Figure 5**). The parental rSG10 caused the following moderate to severe histological changes in all of the sampled tissues: lymphocytic infiltration of the lamina propria (black arrows); submucosal congestion of the tracheal mucosa (empty arrows); hemorrhage, RBC infiltration (black arrows) and congestion (empty arrows) in the lung; severe mucosal epithelial shedding in the proventriculus (empty arrows); congestion of the lamina propria (black arrows); necrosis and shedding of lymphocytic cells in the mucosa of cecal tonsil (empty arrows); increased and gathered microglial cells in the brain (black arrows); and multifocal confluent coagulative necrosis (black arrows) in the spleen (**Figure 5**). However, the chimera rSGLaNPPL exhibited decreased virulence with mild or moderate lesions in the trachea, lung, proventriculus and cecal tonsil, as well as no obvious changes in the brain and spleen compared with the parental rSG10 virus.

**Table 4:** Histology of the organs of inoculated SPF chickens.

Organs	Groups				
	0.9%NaCl	rLaSota	rLaSGNPPL	rSGLaNPPL	rSG10
Trachea	-^a^	-	-	++	+++
Lung	-	-	+	++	++
Proventriculus	-	-	-	+	++
Cecal tonsil	-	-	-	+	+++
Brain	-	-	-	-	++
Spleen	-	-	-	-	+++

*Chickens infected with 0.9%NaCl, rLaSota, rLaSGNPPL, rSGLaNPPL or rSG10 were examined at 4 dpi. ^a^Mean of the severity index from two chicks: -, no change; +, mild; ++, moderate; +++, severe.*

### Viral RNA Synthesis in NDV Minigenome Systems

To further detect whether viral RNA transcription and replication correlated with the intrinsic activity of the polymerase-associated proteins, two NDV minigenome systems (pLaSota-FLuc and pSG10-FLuc) were constructed and a dual luciferase assay was carried out.

**FIGURE 6:**
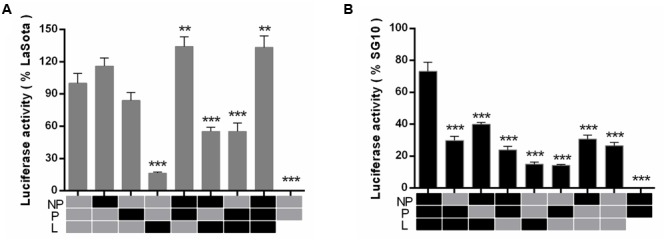
**Relative reporter gene luciferase expression from the minigenome system.** The viral minigenome plasmid pLaSota-FLuc **(A)** or pSG10-FLuc **(B)**, and plasmids encoding NP, P, L of LaSota (gray) or SG10 (black) in various combinations were transfected into BSR-T7 cells in 24-well plates in triplicate and relative luciferase activities were measured as described in Section “Materials and Methods.” The background level of luciferase activity was determined by omitting the L plasmid. Error bars are the standard deviation of the mean (SD). Significance was considered as follows: ^∗∗^*p* = 0.001–0.01, very significant; ^∗∗∗^*p* = 0.0001–0.001, extremely significant.

For the pLaSota-Fluc minigenome, using the SG10 helper plasmids, NP or P proteins individually, no significant differences were observed in comparison (*p* > 0.05) with that of the LaSota replication proteins only. The FLuc activity was reduced in the presence of L, NP + L, and P + L proteins from SG10 (*p* < 0.001). However, the two combinations, NP + P and NP + P + L of SG10, caused a highly significant increase in FLuc activity compared with the native LaSota strain (*p* < 0.01) (**Figure 6A**). For the second pSG10-FLuc minigenome, when we replaced the SG10 helper plasmids, NP, P, L, NP + P, NP + L, P + L, NP + P + L with their LaSota strain counterparts, they showed a similar influence on the expression of the reporter gene, resulting in a significant decrease in replication of the FLuc gene (*p* < 0.001) (**Figure 6B**). These results revealed that the activity of the SG10 replication complex was higher than that of the LaSota replication complex, and the combined action of all three homologous replication proteins was required to reach the highest level of activity. The two minigenomes demonstrated different levels of reporter gene expression when driven by the LaSota replication proteins and those of the SG10 virus. This indicated that the nucleotide differences in the leader or trailer sequences that code for the genomic and antigenomic promoter (63.8% homologous) of LaSota and SG10 had a great influence on the efficiency of RNA synthesis.

## Discussion

While all NDVs belong to a single serotype, there is a wide variation in virulence among different strains. The viral virulence is determined by multiple factors, including its tissue tropism, its ability to deal with the host’s immune system and its efficacy of replication (Rout and Samal, 2008; Zhang et al., 2013). The proteolytic cleavage site of the F protein was shown to be a primary determinant and some other protein genes have also been shown to contribute significantly to NDV virulence (Nagai et al., 1976; Dortmans et al., 2011; Kim et al., 2011; Zhang et al., 2013). However, there are examples of incongruity between different protein genes and virulence, and the contribution of these genes to virulence may be related to the particular NDV strain involved (Estevez et al., 2007; Rout and Samal, 2008; Dortmans et al., 2009, 2010; Wakamatsu et al., 2009; Paldurai et al., 2014). In this study, the velogenic genotype VII strain SG10 and the lentogenic genotype II strain LaSota were chosen to evaluate the roles of the envelope-associated and polymerase-associated protein genes in NDV virulence individually or in combination. The chimeric viruses were examined for their virulence using several different methods, including MDT, ICPI, replication kinetics *in vitro* and *in vivo* and tissue tropism in eight different tissues, as well as clinical symptom observation and mortality in 4-week-old chickens.

The outer surface glycoproteins have been shown to play an important role in the virulence of NDV (Panda et al., 2004; Wakamatsu et al., 2006; Vigerust and Shepherd, 2007; Kim et al., 2011, 2016; Hu et al., 2012). We first confirmed the roles of the F and HN envelope glycoproteins in NDV virulence by reciprocally exchanging them in combination between the velogenic genotype VII strain SG10 and the lentogenic genotype II strain LaSota. When the envelope-associated protein genes (F + HN) of rLaSota were transferred into the rSG10 backbone, the chimeric virus rSGLaFHN exhibited significantly decreased virulence with an ICPI value of 0.86 compared with 1.86 of its parental virus. Conversely, when these genes of rSG10 were transferred into the rLaSota backbone, the chimeric virus rLaSGFHN showed greatly increased virulence with an ICPI value of 1.49 compared with 0.00 of its parental virus. In summary, the exchange of envelope-associated protein genes F and HN together altered the virulence of NDV from velogenic or lentogenic to mesogenic. These results demonstrated that F and HN together play an important role in the virulence of NDV, but that there had to be additional factors affecting the virulence of the virus.

We next evaluated the contributions to genotype VII NDV virulence of the polymerase-associated protein genes (NP, P, and L), there being several examples of incongruity between different protein genes and virulence, as well as evidence that the contribution of these genes to virulence might be related to the particular NDV strain involved (Dortmans et al., 2010; Zhang et al., 2013). When the genes were exchanged alone between the velogenic genotype VII strain rSG10 and the lentogenic genotype II strain rLaSota, no obvious changes in virulence were observed, except for a significant decrease in virulence for rSGLaL. Although the amino acid sequence identity of the NP, P and L genes between rSG10 and rLaSota are low (NP: 91.4%; P: 83%; L: 93.4%), our results demonstrated that the decreased virulence of rSGLaL is not associated with the sequence difference of the L gene and it may be influenced by the interaction among the NP, P, and L proteins. It was somewhat astonishing that the results of the replication kinetics in DF-1 cells showed that the exchange of the NP gene between rLaSota and rSG10 affected the growth of the virus, rSGLaNP exhibiting decreased and rLaSGNP increased replication compared to their parental viruses. Our results thus differed from the findings of other studies which showed that the exchange of the NP gene had no effect on the virus replication kinetics (Rout and Samal, 2008; Paldurai et al., 2014). Further studies are needed to identify the reasons for this. In the present study, more attention has been given to the performance of these genes in combination.

The replacement of the polymerase-associated genes (NP, P, and L) in combination between rLaSota and rSG10 affected the virulence of the chimeric virus, especially when the L gene was involved. For rLaSGNPPL, in which the NP, P, and L genes of the rLaSota virus were replaced with those of the rSG10 virus, the ICPI value was 0.63, which had obviously increased when compared with that of its parental virus rLaSota (0.00). Again, the rSG10 derivatives rSGLaNPL, rSGLaPL and rSGLaNPPL, bearing the polymerase-associated protein genes in combination from rLaSota, shared a decreased virulence with ICPI values 1.48, 1.40, and 1.32, respectively. In addition, the change in the replication kinetics *in vitro* or *in vivo* of each chimeric virus mimicked the change in its virulence, indicating that the change in pathogenicity may be correlated with the changed replication level of the chimeric virus. These findings are in agreement with some previous studies (Dortmans et al., 2010; Paldurai et al., 2014). Our results further indicated that the polymerase L protein of NDV was the next most prominent individual contributor and was sometimes augmented by the homologous NP and P proteins.

We further determined the pathogenicity of two chimeric viruses (rSGLaNPPL and rLaSGNPPL) and their parental viruses in 4-week-old SPF chickens inoculated via a natural infection route. There was a marked difference in the pathogenicity and tissue tropism between the parental viruses and the two chimeric viruses. The NDV strain rSG10 caused a 100% mortality at 5 dpi, and the tissue titers remained at a high level up to the end of the sampling at 5 dpi, whereas the chimeric virus rSGLaNPPL maintained a 0% mortality until 14 dpi and it replicated at a lower level than its parental virus in all sampled tissues. In contrast, the parental rLaSota failed to cause clinical manifestations and the tissue titers were limited in the trachea or occasionally in the duodenum. Its chimeric virus rLaSGNPPL also failed to cause any mortalities, but showed an increased tissue tropism or replication efficiency in some tissues, including trachea, lung, proventriculus, duodenum and brain. The results suggested that exchange of the polymerase-associated genes together (NP + P + L) had a substantial effect on the tissue tropism of the two viruses, which may be associated with the variations of virulence.

It has been reported for NDV that the levels of RNA synthesis are associated with virulence (Madansky and Bratt, 1981). In this study, we further investigated the correlation of viral RNA synthesis with the intrinsic activity of the polymerase-associated proteins using two NDV minigenome systems and a dual luciferase assay. The two minigenomes showed different levels of reporter gene expression when driven by the LaSota replication proteins or those of the SG10 virus, indicating that the nucleotide differences in the leader and trailer sequences had a great influence on the efficiency of the RNA synthesis. However, the roles of these regions in the pathogenesis of NDV have not been investigated. The combination of NP, P and L proteins of SG10 drove higher replication level for both the pSG10-FLuc and the pLaSota-Fluc minigenomes. A possible explanation may be that the polymerase-associated protein of SG10 is inherently more active, and this was dependent on the presence of the homologous interaction partners. The replacement of L protein in the pLaSota-Fluc minigenome resulted in the greatest decrease. The reason behind this phenomenon may be connected with the domains that were required for binding to their respective P proteins or oligomerization differences between the two viruses (Peeters et al., 1999; Huang et al., 2004; Lin et al., 2005), so that the L protein may not function fully in all of its catalytic activities associated with the viral polymerase. The ribonucleoprotein complex (NP, P, and L protein), as the minimum structural unit, is required for viral transcription and replication. These functions are carried out by the polymerase complex in its entirety, not by individual proteins.

In summary, we evaluated the role of the envelope-associated and the polymerase-associated protein genes in NDV virulence individually or in combination by exchanging genes between the prevalent genotype VII strain SG10 and the lentogenic genotype II strain LaSota. Our study further confirmed that the virulence of NDV is determined by multiple viral proteins. The F and HN proteins are the major contributor to virulence, and the L protein as well as NP and P protein also have an obvious influence on virulence. The results have distinct implications in furthering a comprehensive understanding of the virulence factors associated with NDV and other paramyxoviruses. Based on the obtained structure of the mononegavirales L protein (Liang et al., 2015; Paesen et al., 2015), further studies will be carried out to identify the underlying structural and molecular mechanistic basis in the L protein that have an important impact on the virulence.

## Author Contributions

Conceived and designed the experiments: X-hY, J-hJ, and G-zZ. Performed the experiments: X-hY, J-lC, J-hJ, and YS. Analyzed the data: X-hY, JX, JZ, G-zZ. Contributed reagents/materials/ analysis tools: G-zZ. Wrote the paper: X-hY, JZ, and G-zZ.

## Conflict of Interest Statement

The authors declare that the research was conducted in the absence of any commercial or financial relationships that could be construed as a potential conflict of interest.
